# A daily diary study on maladaptive daydreaming, mind wandering, and sleep disturbances: Examining within-person and between-persons relations

**DOI:** 10.1371/journal.pone.0225529

**Published:** 2019-11-27

**Authors:** David Marcusson-Clavertz, Melina West, Oscar N. E. Kjell, Eli Somer

**Affiliations:** 1 Department of Psychology, Lund University, Lund, Sweden; 2 Department of Psychology, Ludwig Maximilian University of Munich, Munich, Germany; 3 Department of Psychological Sciences, University of Connecticut, Connecticut, United States of America; 4 School of Social Work, University of Haifa, Israel; Radboudumc, NETHERLANDS

## Abstract

Cross-sectional and experimental research have shown that task-unrelated thoughts (i.e., mind wandering) relate to sleep disturbances, but there is little research on whether this association generalizes to the day-level and other kinds of task-unrelated mentation. We employed a longitudinal daily diary design to examine the within-person and between-person associations between three self-report instruments measuring mind wandering, maladaptive daydreaming (a condition characterized by having elaborate fantasy daydreams so insistent that they interfere with daily functioning) and sleep disturbances. A final sample of 126 participants self-identified as experiencing maladaptive daydreaming completed up to 8 consecutive daily reports (in total 869 daily observations). The scales showed acceptable-to-excellent within-person reliability (i.e., systematic day-to-day change) and excellent between-person reliability. The proportion of between-person variance was 36% for sleep disturbances, 57% for mind wandering, and 75% for maladaptive daydreaming, respectively (the remaining being stochastic and systematic within-person variance). Contrary to our pre-registered hypothesis, maladaptive daydreaming did not significantly predict sleep disturbances the following night, *B* = -0.00 (*SE* = 0.04), *p* = .956. Exploratory analyses indicated that while nightly sleep disturbances predicted mind wandering the following day, *B* = 0.20 (*SE* = 0.04), *p* < .001, it did not significantly predict maladaptive daydreaming the following day, *B* = -0.04 (*SE* = 0.05), *p* = .452. Moreover, daily mind wandering did not significantly predict sleep disturbances the following night, *B* = 0.02 (*SE* = 0.05), *p* = .731. All variables correlated at the between-person level. We discuss the implications concerning the differences between maladaptive daydreaming and mind wandering and the possibility of targeting sleep for mind wandering interventions.

## Introduction

People spend a large amount of their waking time mentally drifting away to subjects unrelated to what they are doing at the moment (mind wandering) [1]. Those who mind wander to a greater degree are more likely to experience sleep difficulties [2], but recent research have highlighted the heterogeneity of mind wandering features [3] and the importance of acknowledging that not all task-unrelated mentations relate equivalently to psychological functioning [4]. A type of mentation termed maladaptive daydreaming is characterized by elaborate narratives with high degree of fancifulness, vividness, compulsion, and interference with daily life functioning [5, 6]. Maladaptive daydreaming is not only temporally associated with adverse outcomes, including obsessive-compulsive and anxiety symptoms [7], but is also associated with a high comorbidity rate with a number of psychiatric disorders, including anxiety and obsessive-compulsive disorders [8]. Maladaptive daydreaming is distinct from general mind wandering mainly because it focuses on structured, intentionally-generated fantasy narratives, whereas mind wandering is broader and includes fleeting thoughts and imagery that seemingly come up spontaneously and relate to the person’s daily life, although some conceptualizations of mind wandering also includes guided thoughts [3]. In this study we aimed to examine how sleep relates to mind wandering and maladaptive daydreaming, both within- and between-persons, using a longitudinal design.

### Mind wandering and sleep

Poor sleep is associated with adverse health outcomes, including greater mortality hazard [9] and risk for coronary heart disease [10]. Despite the importance of sleep, the recommended level of 7 hours per night for adults [11] is often not met. For instance, nearly one-third of American adults fail to reach even 6 hours of sleep per night [12], which suggests a need to improve our understanding of the antecedents (and consequences) of sleep behaviors. The association between poor sleep and proneness to lapses in attention is well-documented [13], but there is less research on the relation between sleep and internal distractions, such as mind wandering. Among the few exceptions is a multi-study survey, which indicated that individual differences in mind wandering is associated with sleep disturbances [2]. The study employed mediational modeling to indicate a bidirectional relation between mind wandering and sleep quality. An experimental study indicated greater amount of mind wandering in a visual search task for the group with one night of total sleep deprivation, compared to the restful-wakefulness group—but this effect was observed only during high perceptual load [14]. Another study found an association between daily life mind wandering (measured with experience sampling during one day) and difficulty falling asleep (measured with a single questionnaire item [15]). However, neither of these studies employed repeated sampling across days to examine within-person relations between these variables, making it difficult to understand how these associations unfold over time.

### Maladaptive daydreaming and sleep

There is paucity of research on the relation between maladaptive daydreaming and sleep, but there is some indirect evidence pointing to a relation between the two. First, one study included an item on difficulty falling asleep in a three-item self-diagnostic tool for maladaptive daydreaming which predicted the standard measure of maladaptive daydreaming [6]. Second, dissociation is a related construct that includes absorption to inner experiences and shows medium-to-large associations with sleep disorders [16] and a large association with maladaptive daydreaming [17]. Third, the potentially related constructs of Guilty-Dysphoric and Anxious-Distractible daydreaming styles have been shown to relate to sleep disturbances [18]. However, maladaptive daydreaming plots appear to be constructed in an attempt to satisfy deep psychological needs and are reported to be immensely rewarding, thus creating a yearning to repeatedly engage in long-lasting episodes of daydreaming [19]. These episodes could interfere with health-related behaviors such as sleeping. Personal communications with the fourth author and online discussions in maladaptive daydreaming communities attested to the loss of sleep associated with the urge to daydream [20]. To further support this claim, sleep disturbances are found to be related to attention deficit hyperactivity disorder (ADHD), obsessive compulsive disorder (OCD), and other difficulties with attention and repetitive thinking or rumination [21–24]). All of these conditions have a high comorbidity and overlapping symptoms with maladaptive daydreaming [8].

### The present study

Our primary aim was to examine how maladaptive daydreaming and mind wandering during the day relate to sleep on the previous and upcoming nights, while also accounting for person-level relations. We employed a daily diary design in which volunteers completed up to eight daily surveys including measures of mind wandering, maladaptive daydreaming, and perceived sleep quality. Our pre-registered hypothesis was that maladaptive daydreaming on the day would predict poor sleep quality the upcoming night, statistically controlling for mind wandering. We further expected mind wandering to relate to sleep quality at the day-level. We also expected that mind wandering and maladaptive daydreaming would predict poor sleep quality at the person level.

## Method

### Participants

A total of 152 individuals participated in the study. Participants were recruited via invitations sent to a list of individuals who had previously contacted the last author indicating their desire to be invited to participate in studies on maladaptive daydreaming. These individuals were self-identified maladaptive daydreamers who wished to inform researchers in this area about their experiences, seek advice, and encourage further research [20]. Recruitment was, therefore, targeted to include higher rates of maladaptive daydreaming in our sample than the general population. To be eligible for participation, individuals were required to be over 18 years of age and be proficient in reading and understanding English. Participants had a mean age of 30.04 (*SD* = 10.65; range = 18–73) years. There were 118 females, 30 males, and 4 who did not identify themselves as male nor female. Participants were from USA (43%), England (14%), India (6%), Canada (6%), Australia (5%), and 30 other countries (29%). The reported socioeconomic status mean was 4.8 (*SD* = 1.83) on the 1–9 socioeconomic ladder scale. Sixty-six participants reported one or more psychiatric diagnoses, of which 38 reported Depression, 27 Anxiety, 10 OCD, 5 ADHD, 6 Post-Traumatic Stress Disorder, 5 Bipolar Disorder, among other diagnoses. Thirty-two reported being in therapy; 34 that they were on medication; and 25 reported taking some kind of sleep medication. Previous cross-sectional research has found correlations above *r* = .30 between mind wandering and global measures of sleep difficulties [2], which would require a sample of at least 84 people for 80% power.

### Materials

#### The 16-item Maladaptive Daydreaming Scale (MDS-16)

The MDS-16 is a self-report measure of maladaptive daydreaming [6] answered using an 11-point scale. A recent revision of the original 14-item scale includes 16 items, and has shown high internal consistency [25]. A confirmatory analysis on the Arabic version of the MDS-16 showed good fit for a 2-factor solution [25]. The first subscale comprises 9 items loading on a factor labelled “Immersive daydreaming” with a sample item being “How often are your current daydreams accompanied by vocal noises or facial expressions (e.g. laughing, talking or mouthing the words)?”. The second subscale comprises 7 items loading on a factor labelled “Distress and impairment” with a sample item being “Some people have the experience of their daydreaming interfering with their daily chores or tasks. How much does your daydreaming interfere with your ability to get basic chores accomplished?”. We used an adapted daily diary version of the questionnaire [7] with the following instructions:

In answering the following questions, please refer to your daydreaming activities today. Choose the option that best fits your experience: “0” states that the experience did not happen today, while “1” through “10” state the intensity of the experience if it did occur.

For example, item #2 was rephrased as: “I felt the need or urge to continue a daydream, that was interrupted by a real-world event, at a later point”. Cronbach’s alpha for Day 0 was .92 in the previous study [7], indicating high reliability at the start of the study. Scores for each item are averaged for an overall daily score. A cutoff of 50% (or a score of 5 in the current study) has been identified to best distinguish those with clinical level maladaptive daydreaming from non-maladaptive daydreamers on the trait version of the MDS-16 [25].

#### Mind Wandering Questionnaire (MWQ)

This trait-like questionnaire measures overall mind wandering tendencies with 5 items. We adapted it to a daily diary design (one item was excluded because it was not suitable for a daily diary design). All items were answered on a 6-point scale, with participants indicating how often they had each experience TODAY (*almost never; very infrequently; somewhat infrequently; somewhat frequently; very frequently; almost always*). A sample item is “I found myself listening with one ear, thinking about something else at the same time”. The trait-like questionnaire has shown high internal consistency and a principal component analysis indicated one factor with eigenvalue above one with that factor explaining about 63.16% of the total variance. The scale also moderately predicted mind wandering as measured with thought sampling [26].

#### Patient-Reported Outcomes Measurement Information System (PROMIS) Sleep disturbance scale short form

The short form of the PROMIS sleep disturbances scale comprises 8 items about sleep difficulties, including difficulty falling asleep, staying asleep, and overall sleep quality. A sample item is “Last night… my sleep was restless” answered on a Likert scale; 1 (*Not at all)*, *2 (A little bit)*, *3 (Somewhat)*, *4 (Quite a bit)*, and *5 (Very much*). A study provided support for its validity and reliability and showed that the short form provides better measurement precision than standard sleep quality questionnaires despite having fewer items [27]. The scale has also shown moderately high correlations (*r*_s_ = -.46, *p* = .02) with total sleep time measured with actigraphy [28]. Nightly sleep disturbances are indexed by taking the mean of all 8 responses.

#### Control items

We added one control item for each of the eight daily surveys, presented within the MWQ. The control item prompted participants to “please select option 3 ‘somewhat infrequently’ here”. Participants that answered more than one of these items inaccurately were excluded from all analyses (pre-registered cut-off). Attention checks similar to this method have been shown to increase reliability and power [29].

### Procedure

Participants were first informed about the study and indicated their consent online (written form). The internet-based survey was constructed and administered using Qualtrics Survey Software. The study commenced on a set date for all participants and proceeded for eight consecutive days. Each day, participants were sent the survey link via email and were instructed to complete the survey after 6:00 PM their local time. A second reminder email was sent each day 14 hours after the initial email in order to account for various time zone differences. The reason for this was so that the first email was sent at 6pm for participants in the earliest time zone (AEST/UTC+10), and the second email was sent at 6pm for participants in the latest time zone (EST/UTC-4), based on the location information of those in our sample. Both emails were sent to all participants so that they would also function as a reminder. In each daily survey, participants were required to enter their local time and day. Demographic information was collected on the first day, including age, gender, country of residence, years of education, socio-economic status, mental health status, and medication use. Each day, participants completed the daily MDS-16, MWQ, and PROMIS in a random order. The study was approved by the Ethics Committee of the Faculty of Social Welfare and Health Sciences at University of Haifa (case 399/18).

### Analyses

We conducted three multilevel models with the daily reports of maladaptive daydreaming, mind wandering, and sleep disturbances as outcomes, respectively. In these models, daily observations were designated as level-1 (within-person) and individual variables were designated as level-2 (between-person, including baseline measures and person mean scores of daily measures). Each level-1 predictor was within-person centered so that a positive score means higher than the person’s own average across the whole study period. In the model with sleep disturbances as outcome, we lagged the predictors (mind wandering, maladaptive daydreaming) one day, so that we analyzed whether the latter predicted sleep the following night. Robust (empirical) standard errors and maximum likelihood estimation was used as pre-registered (https://aspredicted.org/ud7fr.pdf). We did not pre-register random vs. fixed effects or variance-covariance structure but employed random intercepts and fixed slopes and unstructured variance covariance matrices for all analyses. In support of employing random intercept modeling, there was significant between-person variance in each model. All models converged within three iterations. The analyses were performed with SAS 9.4 (proc mixed). We concluded that normality assumptions were met based on visual inspection of the residual plots. We report unstandardized *B* coefficients with *SE*s in parentheses.

For maladaptive daydreaming as outcome, we also explored a model that included two-way interactions between maladaptive daydreaming and sleep disturbances (as pre-registered). For that model we created standardized predictors at level-1 by dividing the within-person centered scores with the within-person SDs and at level-2 by dividing the grand-mean centered person means with the grand SDs. We used the raw scores in all other models. A sample SAS model follows (MWQ as the outcome, PROMIS sleep disturbances and MDS as the predictors):

**proc mixed** data = Mds_sleep_mwq covtest method = ml empirical plots = ALL;

class ID;

model MWQ_day = PROMIS_day_within_person_centered

                     MDS_day_within_person_centered

                     PROMIS_day_personmean

                     MDS_day_personmean

                                        /solution ddfm = bw;

random intercept /subject = ID type = un g gcorr;

**run**;

We decomposed daily diary variance into between-person and within-person variance and estimated reliability within-person and between-person [30]. The within-person reliability measures systematic changes from day to day. We applied the SAS script (https://osf.io/y3mfe/) used in previous research [31]. We performed robust data visualization using raincloud plots in R [32].

## Results

### Descriptive summary

We first checked the accuracy on the daily control items and the compliance rate of daily diary reports. In summary, 144 out of 152 (95%) individuals passed control items screening. As for compliance rate, 133 out of 152 people (88%) completed at least three days of data, which were required for the within-person analyses. Taking both issues into account, 126 out of 152 (83%) individuals passed both cutoffs, and the remaining analyses are based on those 126 individuals. They contributed with an average of 6.89 (*SD* = 1.63) daily reports, which corresponds to a mean compliance rate of 86%. Compliance rate did not correlate significantly with persons’ mean scores on any of the three scales (maladaptive daydreaming, mind wandering, sleep disturbances, *p*s greater than .2).

Approximately half of the full sample (48%) reported maladaptive daydreaming at the clinical level (i.e., MDS score above 5) with MDS scores averaged across the 8 days (*M* = 4.97, range = 0.42–9.29). This suggests that a substantial portion of the sample were likely to be experiencing severe maladaptive daydreaming symptoms. Indeed, when inspecting each individual’s highest daily MDS score, 72% of the sample scored above the clinical level, suggesting that a vast majority of the sample had at least one day characterized by severe maladaptive daydreaming.

**Fig 1:**
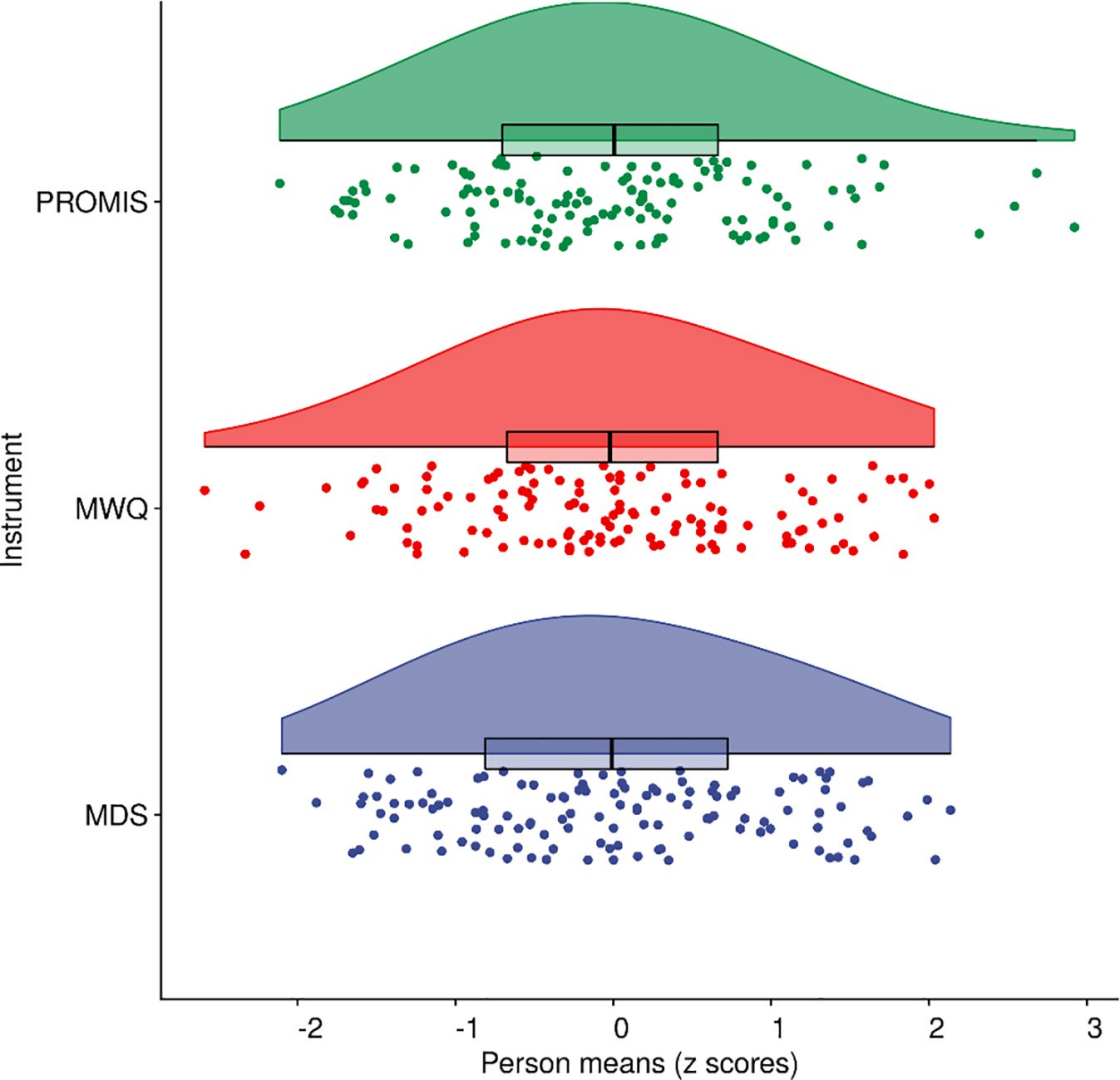
Raincloud plot of person mean scores (*z*) for each instrument administered across eight days. (MDS = Maladaptive daydreaming scale; MWQ = Mind wandering questionnaire; PROMIS = Patient-reported outcomes measurement information system–Sleep disturbances short form). Each dot shows the observed person mean score for that instrument (jittered). The lower and upper hinges on the boxplots represent the first and third quartile with the median in between, whereas the whisker lines extend to the most extreme values but no further than 1.5 × the interquartile range from the hinge. The raincloud shapes illustrate the shapes of the distributions based on Kernel density probability functions (scaled to have equal heights across instruments).

We next checked the psychometric properties of the three scales before analyzing their relations. Table 1 shows aggregate and multilevel descriptive statistics for the three scales. Each scale showed near-perfect between-person reliability (> .98) across the 8-day study period. Within-person reliabilities ranged from acceptable (the MWQ) to good or excellent (the MDS and the PROMIS), indicating that each scale could detect systematic changes from day to day. Intraclass correlation coefficients from the null model of each variable indicated that 75% of variance in maladaptive daydreaming scores (MDS) was between individuals (i.e., 25% of MDS variance was within people, which include both systematic and error variance). Approximately 57% of the variance in mind wandering was between-individuals, whereas 36% of the variance in PROMIS sleep disturbances was between-individuals. In other words, sleep disturbances fluctuated highly from day to day, whereas maladaptive daydreaming was relatively stable. The skewness was close to zero for person means of each instrument, indicating quite symmetric distributions (Fig 1).

**Table 1:** Descriptive summary of research instruments measured daily over 8 Days (= 126 Individuals & 869 Observations).

Scale	Aggregated			Multilevel				
	*M*	*SD*	Skew	Variance		Reliability		ICC
				Between	Within	Between	Within	
PROMIS Sleep disturbances	2.76	0.71	0.36	0.40	0.71	.98	.93	.36
Mind wandering questionnaire	3.96	0.97	-0.04	0.85	0.66	.98	.76	.57
Maladaptive daydreaming scale	4.82	2.09	0.12	4.11	1.38	.99	.85	.75

*Note*: Intraclass correlation coefficients were computed from null models in SAS.

### Correlations between maladaptive daydreaming, mind wandering, and perceived sleep disturbances

**Fig 2:**
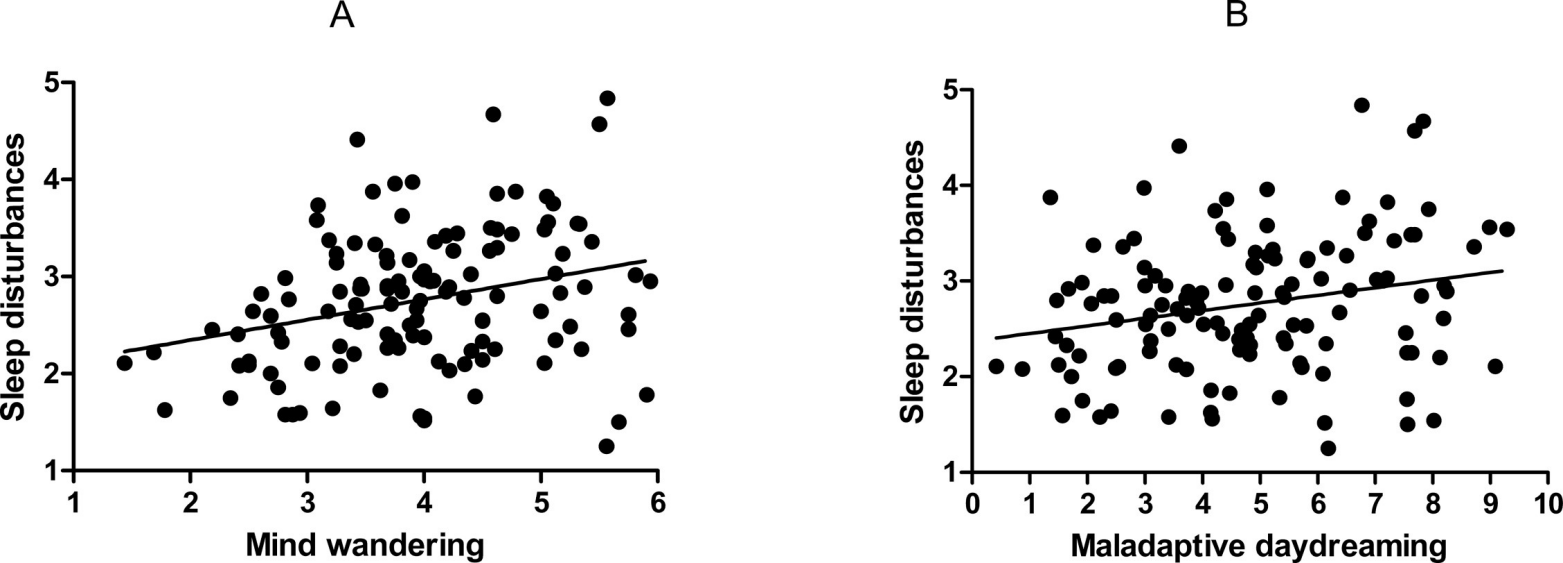
**Scatterplots of sleep disturbances regressed on (A) mind wandering, and (B) maladaptive daydreaming.** Scores are aggregated to person level so that each observation represents person mean score.

We first evaluated the Pearson correlations between these measures at between-person and within-person level. At the between-person level, there was a large correlation between maladaptive daydreaming and mind wandering, *r*(124) = .66, *p* ≤ .001, and small-to-medium correlations between sleep disturbances and mind wandering, *r*(124) = .29, *p* = .001, and sleep disturbances and maladaptive daydreaming, *r*(124) = .23, *p* = .008. That is, individuals with greater sleep disturbances report more mind wandering and maladaptive daydreaming in overall (Fig 2).

At the within-person level, days with greater maladaptive daydreaming than usual were associated with greater mind wandering, *r*(774) = .38, *p* ≤ .001. However, days with greater maladaptive daydreaming than usual were not significantly associated with sleep disturbances on the following night, *r*(699) = .00, *p* = 1.0. Similarly, days with greater mind wandering than usual were not significantly associated with sleep disturbances on the following night, *r*(699) = .02, *p* = .607.

### Multilevel analyses with perceived sleep disturbances as outcome

Individuals on sleep medication did not report significantly different sleep disturbances compared to those who did not take sleep medication, *B* = 0.17 (*SE* = 0.17), *t*(124) = 1.01, *p* = .315). Medication was not included further as a covariate.

We next tested the hypothesis that maladaptive daydreaming would predict sleep disturbances the following night, statistically controlling for mind wandering in the model (and including both within- and between-person effects). The results did not support this hypothesis. As shown in Table 2, the only significant predictor of sleep disturbances was mind wandering at the between-person level. That is, those individuals who reported greater mind wandering in general reported greater sleep disturbances. Specifically, a one-unit increase on the MWQ was associated with a 0.19 unit increase on the PROMIS sleep disturbances scale.

**Table 2:** Multilevel Analyses with Mind wandering and Maladaptive daydreaming last day as predictors of Sleep Disturbances that Night.

Fixed effect	*B*	*SE*	*df*	*t*	*P*
Intercept	1.82	0.26	122	7.09	< .001***
Maladaptive daydreaming, within-person	-0.00	0.04	572	-0.06	.956
Mind wandering, within-person	0.02	0.05	572	0.34	.731
Maladaptive daydreaming, between-person	0.04	0.04	122	0.93	.352
Mind wandering, between-person	0.19	0.09	122	2.02	.046*

*Note*: Sleep disturbances were taken from current day and referring to last night’s sleep, whereas mind wandering and maladaptive daydreaming were taken from the previous day (i.e., last day’s report of mind wandering or maladaptive daydreaming that day).

As the between-person association between mind wandering and maladaptive daydreaming was large (i.e., a moderate variance inflation factor of 1.78 in an ordinary least squares regression) it is worth noting that if mind wandering was dropped from the model, maladaptive daydreaming predicted sleep disturbances, *B* = 0.10 (0.03), *t*(123) = 2.99, *p* = .003 (between-person). Likewise, if maladaptive daydreaming was dropped from the model, mind wandering predicted sleep disturbances, *B* = 0.24 (0.07), *t*(123) = 3.55, *p* < .001 (between-person). In sum, although person means of mind wandering and maladaptive daydreaming predicted sleep disturbances when analyzed separately, only mind wandering predicted sleep disturbances with both predictors included in the model. Next, we analyzed other temporal relations with maladaptive daydreaming as the outcome, which we pre-registered as a secondary analysis.

### Multilevel analyses with maladaptive daydreaming as outcome

Table 3 shows the results with daily maladaptive daydreaming as the outcome with sleep disturbances and mind wandering as predictors. All these measures were taken from the same observation (i.e., not lagged) as sleep referred to last night’s sleep. As shown in Table 3, mind wandering predicted maladaptive daydreaming both within- and between-person, but sleep disturbances did not significantly predict maladaptive daydreaming. If mind wandering was dropped from the model, then sleep disturbances predicted maladaptive daydreaming but only at the between-person level, *B* = 0.69 (0.26), *t*(124) = 2.65, *p* = .009.

**Table 3:** Multilevel analyses with Mind wandering today and sleep last night as predictors of Maladaptive daydreaming today.

Fixed effect	*B*	*SE*	*df*	*t*	*P*
Intercept	-1.07	0.68	123	-1.57	.119
Sleep disturbances, within-person	-0.04	0.05	740	-0.75	.452
Mind wandering, within-person	0.55	0.08	740	7.00	< .001***
Sleep disturbances, between-person	0.15	0.20	123	0.72	.475
Mind wandering, between-person	1.39	0.13	123	10.37	< .001***

*Note*: All day-level measures were taken from the same day observation so that sleep referred to last night and maladaptive daydreaming and mind wandering referred to current day.

Following our pre-registration, we also explored interactions between mind wandering and sleep disturbances on maladaptive daydreaming by adding two interaction terms (within- and between- person) to the model. First, the simple effects of mind wandering within- and between-person remained significant as predictors of maladaptive daydreaming (*p*s < .001). Furthermore, the within-person interaction between sleep disturbances and mind wandering was significant, *B* = -0.11 (0.05), *t*(739) = -2.19, *p* = .029, indicating that the positive relation between daily mind wandering and maladaptive daydreaming was reduced following a poor night’s sleep. The between-person interaction was not significant, *B* = 0.01 (0.12), *t*(122) = 0.11, *p* = .915.

### Multilevel analyses with mind wandering as outcome

In the final analysis we explored mind wandering as the outcome with sleep disturbances and maladaptive daydreaming from the same observation as predictors (Table 4). At the within-person level, greater sleep disturbances predicted increased mind wandering the following day. Greater maladaptive daydreaming was associated with mind wandering on the same day. At the between-person level, those with greater maladaptive daydreaming in general reported more mind wandering.

**Table 4:** Multilevel analyses with maladaptive daydreaming today and sleep last night as predictors of Mind wandering today.

Fixed effect	*B*	*SE*	*df*	*t*	*p*
Intercept	2.01	0.27	123	7.48	< .001***
Sleep disturbances, within-person	0.20	0.04	740	5.46	< .001***
Maladaptive daydreaming, within-person	0.25	0.03	740	8.07	< .001***
Sleep disturbances, between-person	0.20	0.11	123	1.86	.065
Maladaptive daydreaming, between-person	0.29	0.03	123	8.99	< .001***

*Note*: All day-level measures were taken from the same day observation so that sleep referred to last night and maladaptive daydreaming and mind wandering referred to current day.

## Discussion

To sum up, we examined the day-to-day and between-person associations between maladaptive daydreaming, mind wandering, and sleep disturbances using self-report data across eight days. We found the following: First, the daily diary versions of the scales showed acceptable-to-excellent within-person reliability and excellent between-person reliability. Second, mind wandering and maladaptive daydreaming exhibited a medium association within-person (14% shared variance) and large between-person (44% shared variance). Third, contrary to our hypothesis, maladaptive daydreaming on the current day did not predict sleep disturbances the following night. Fourth, although maladaptive daydreaming and mind wandering correlated with sleep disturbances between-person, only mind wandering significantly predicted sleep disturbances with both predictors in the model. Fifth, although sleep disturbances predicted mind wandering on the following day, it did not significantly predict maladaptive daydreaming.

Contrary to our expectations, we did not find evidence for daily mind wandering nor maladaptive daydreaming predicting the forthcoming night’s sleep quality. The associations were close to zero and non-significant regardless of whether either or both predictors were included in the model and despite the high within-person reliability of these measures. One limitation of this finding is that we measured these three constructs once per day (after 6 pm). In order to avoid a compromised timing of data collection, it would have been better to measure sleep earlier in the day and mind wandering and maladaptive daydreaming later in the day. It is possible that mind wandering or maladaptive daydreaming close to bed time is predictive of subsequent sleep, at least insofar as they keep the mind highly activated (e.g., intensive worrying thoughts) and increase pre-sleep arousal, which predicts sleep [cf., 33]. Another issue to consider is that there was relatively little variance at the day-level for maladaptive daydreaming. One interpretation is that maladaptive daydreaming is more trait-like rather than a state-like construct. Additionally, maladaptive daydreaming could vary across other time periods (e.g., weeks or months). We cannot draw inferences about variance at those levels from this study.

The between-person results indicated that both maladaptive daydreaming and mind wandering were associated with sleep disturbances, as well as strongly related to each other. However, with maladaptive daydreaming and mind wandering included in the same model, only mind wandering related to sleep disturbances. This finding extends previous cross-sectional survey research studies showing that mind wandering and sleep disturbances relate between-persons [2] and experimental work demonstrating that a night of sleep deprivation leads to more mind wandering during a high-load perceptual task [14]. Moreover, maladaptive daydreaming appears to be related to sleep disturbances only via shared variance with mind wandering. This finding implies that mind wandering habits should be considered in regard to reports of sleep disturbances by those suffering from maladaptive daydreaming [6]. The sensitivity to both between- and within-person effects in the current study design highlights the nuances in understanding the relations between these constructs. An additional strength of this longitudinal daily diary design is that sleep was measured in people’s natural settings relatively close to the referent episode, but a welcome addition would be to use even more intensive assessments within the day to mitigate the risk of long-term memory biases affecting the reports.

Exploratory analyses indicated that sleep disturbances predicted mind wandering on the following day, but the former did not significantly predict maladaptive daydreaming. One possible explanation for this distinction is that poor sleep temporarily reduces executive cognitive control, which according to the cognitive failures hypothesis would reduce people’s ability to prevent the mind from wandering [cf., the cognitive failures hypothesis of mind wandering; 34]. Another speculative possibility is that poor sleep leads to lower functional connectivity [35, 36] and greater independence of semantic networks and generation of novel associations [37], which could lead to greater decoherence in the stream-of-consciousness—thus increasing the frequency of mind wandering episodes. Maladaptive daydreaming, on the other hand, may involve a narrower focus of attention, and therefore demand a higher degree of cognitive control and coherence than mind wandering. Future research could test these hypotheses by adding neurocognitive measures to the daily diary design. The implications of sleep predicting mind wandering could be important for designing effective interventions on mind wandering. There has been an upsurge in research focusing on mind wandering interventions to mitigate some of the presumed consequences, such as driving accidents [38] or poor learning in educational settings [39]. Our within-person finding suggests that sleep may be an effective target for mind wandering research, but confirmatory research is needed to verify this finding.

Research on dissociative conditions and sleep could help to explain the lack of association between maladaptive daydreaming and sleep in either direction in the present study. One polysomnography study found that dissociative individuals had a greater proportion of rapid-eye movement sleep during the sleep period and reported more unusual dream-related experiences [40]. As maladaptive daydreaming is highly related to dissociative conditions [17], it may have similar relations to sleep, rather than affecting sleep in the way that ADHD and OCD does. That is, maladaptive daydreaming, while not associated with overall sleep duration, may be associated with other unusual sleep experiences like elaborate dreams, lucid dreaming, and sleep paralysis. Although the examination of such experiences was beyond the scope of the current study, we recommend that future research explore this possibility.

Some limitations of the current study are important to acknowledge. All three scales relied on self-reports, which may be confounded by common method bias and self-report biases (e.g., metacognitive beliefs) [41]. In addition, the day-level questionnaires measuring mind wandering and maladaptive daydreaming were adapted from person-level questionnaires. This adaptation could inflate our estimates of between-person variability. For instance, maladaptive daydreaming exhibited 75% between-person variance, although it could also reflect a highly stable construct. Developing experience sampling questionnaires of maladaptive daydreaming and administering them intensively within days could shed more light on this issue. On the other hand, the low number of study days, other than limiting statistical power, may also obscure some within-person effects of longer durations (e.g., vacation, starting a new job, seasonal effects). In support of our measures, we conceptually replicated the mind wandering-sleep association despite using a different approach to the other studies, one of which measured mind wandering with thoughts probes in the laboratory and experimentally manipulated sleep [14]. In addition, reports from mind wandering questionnaires and thought probes show similar associations to behavioral performance [42] and neural activity [43], while only showing weak associations with measures of social desirability tendencies [44].

Another limitation is that although we discriminated between mind wandering and maladaptive daydreaming, we did not evaluate other aspects of mentation, such as guided versus unguided thoughts, or mind wandering in particular contexts (e.g., high-demanding vs. low demanding tasks). Caution should be taken against generalizing these findings to all contexts [45] and other mind wandering related features [3]. A further limitation is that about 12% of the participants contributed less than three daily reports which prevented within-person analyses on this data. Among the final sample there was 14% of missing daily reports. Our findings should not be generalized to these occasions. Also, with longer study period, the temporal relations between these constructs could be further clarified, such as by investigating the cumulative impact of several adjacent nights of poor sleep on daily mentation.

In sum, results from this pre-registered daily diary study did not yield support for the hypothesis that maladaptive daydreaming during the day predicts sleep the following night. Neither did we find evidence for mind wandering predicting sleep on a daily basis. However, exploratory analyses indicated that sleep disturbances predicted mind wandering on the following day. Sleep and mind wandering were also related between-persons. These findings are consistent with previous cross-sectional research on mind wandering and sleep. Taken together, we conclude that there is converging evidence suggesting that sleep disturbances or deprivation predict mind wandering, but there is no such evidence for sleep predicting maladaptive daydreaming, which may be interpreted to reflect divergence of mind wandering and maladaptive daydreaming (although caution must be taken against interpreting null results). While there are many anecdotal reports of individuals presenting both maladaptive daydreaming and sleep disturbances, the former is currently understudied and not well understood. The current research addresses an important gap in the literature in suggesting that sleep disturbances are more closely linked with mind wandering than with maladaptive daydreaming. Further enquiry and interventions in maladaptive daydreaming should consider the role of mind wandering as a highly associated, but separate construct that demonstrates sensitivity to sleep disturbance. Additionally, future applied research aiming to intervene in mind wandering should consider sleep disturbances as a potential target.

Our study of the relations between maladaptive daydreaming and mind wandering and sleep yielded three incidental but potentially important findings. First, maladaptive daydreaming appeared to be related to sleep disturbances only via shared variance with mind wandering, but future research designed to test mediational models would be needed to verify this interpretation. Second, mind wandering and maladaptive daydreaming were strongly associated with each other, but less so following a night with sleep disturbances. Third, sleep disturbances predicted daily mind wandering, which may reflect diffuse mode thinking (e.g., fleeting, unguided thoughts), whereas it did not significantly predict maladaptive daydreaming, which could be regarded as a pleasant “focus mode” mental activity, thus, requiring the investment of considerable cognitive resources.

## Supporting information

S1 File SPSS data file. (SAV)

S2 File CSV data file. (CSV)

S3 File Codebook PDF file. (PDF)
