# Supplementary material for: A daily diary study on maladaptive daydreaming, mind wandering, and sleep disturbances: Examining within-person and between-persons relations
Source: PLoS One. 2019 Nov 27;14(11):e0225529. doi: 10.1371/journal.pone.0225529 (PMC6880993; doi:10.1371/journal.pone.0225529)
Supplement: S3 File — (PDF) [file pone.0225529.s003.pdf]

Variable	Name	Label	Values	Code
1	ID	ID	None	
2	studyday	Studyday	None	
3	Agebinned	Age (Binned)	{1, <= 20} {2, 21 - 31} {3, 32 - 41} {4, => 42}	
4	SES_Ladder	SES (1-9)	None	
5	GENDER	Gender	(1=male 2=female 3=other)	
6	Control_day	**D1CONTROL QUESTION (option 3)	None	
7	error_control	error control	(1=error)	
8	controlitemerror_sum	Control Item Error Sum	None	
9	error_pm	Control Item Error person mean	None	
10	Mentalillnessstatus1yes2no	Mental illness status (1=yes 2=no)	{1, yes} {2, No}	
11	ReceivingTherapy1yes2no	Receiving Therapy (1=yes 2=no)	{1, yes} {2, No}	
12	Takingmedication1yes2no	Taking medication (1=yes 2=no)	{1, yes} {2, No}	
13	sleepmedication	Taking sleep medication (1=yes 0=no)	{0, No} {1, Yes}	
14	Index1	Studyday	0-10	
15	MDS1	MDS1	0-10	
16	MDS2	MDS2	0-10	
17	MDS3	MDS3	0-10	
18	MDS4	MDS4	0-10	
19	MDS5	MDS5	0-10	
20	MDS6	MDS6	0-10	

21	MDS7	MDS7	0-10	
22	MDS8	MDS8	0-10	
23	MDS9	MDS9	0-10	
24	MDS10	MDS10	0-10	
25	MDS11	MDS11	0-10	
26	MDS12	MDS12	0-10	
27	MDS13	MDS13	0-10	
28	MDS14	MDS14	0-10	
29	MDS15	MDS15	0-10	
30	MDS16	MDS16	0-10	
31	MWQ1	MWQ1	1-6	
32	MWQ2	MWQ2	1-6	
33	MWQ3	MWQ3	1-6	
34	MWQ4	MWQ4	1-6	
35	PROMIS1	PROMIS1	1-5	
36	PROMIS2	PROMIS2	1-5	
37	PROMIS3	PROMIS3	1-5	
38	PROMIS4	PROMIS4	1-5	
39	PROMIS5	PROMIS5	1-5	
40	PROMIS6	PROMIS6	1-5	
41	PROMIS7	PROMIS7	1-5	
42	PROMIS8	PROMIS8	1-5	
43	MDS_day_mean	MDS_day_mean	None	
44	MWQ_day_mean	MWQ_day_mean	None	
45	PROMIS_day_mean	PROMIS_day_mean	None	
46	MDS_day_pm	MDS Person Mean	None	mean(MDS_day_mean) [by ID]= MDS_day_pm
47	MWQ_day_pm	MWQ Person Mean	None	mean(MWQ_day_mean) [by ID]= MWQ_day_pm
48	PROMIS_day_pm	PROMIS Person Mean	None	mean(PROMIS_day_mean) [by ID]= PROMIS_day_pm
49	MDS_day_sd	MDS Person SD	None	stddev(MDS_day_mean) [by ID] =MDS_day_sd
50	MWQ_day_sd	MWQ Person SD	None	stddev(MWQ_day_mean) [by ID]=MWQ_day_sd

51	PROMIS_day_sd	PROMIS Person SD	None	stddev(PROMIS_day_mean) [by ID]=PROMIS_day_sd
52	MDS_day_pm_gm	MDS Grand Mean	None	mean(MDS_day_pm)= MDS_day_pm_gm
53	MWQ_day_pm_gm	MWQ Grand Mean	None	mean(MWQ_day_pm)= MWQ_day_pm_gm
54	PROMIS_day_pm_gm	PROMIS Grand Mean	None	mean(PROMIS_day_pm)= PROMIS_day_pm_gm
55	MDS_day_pm_gsd	MDS Grand SD	None	stddev(MDS_day_pm)= MDS_day_pm_gsd
56	MWQ_day_pm_gsd	MWQ Grand SD	None	stddev(MWQ_day_pm)= MWQ_day_pm_gsd
57	PROMIS_day_pm_gsd	PROMIS Grand SD	None	stddev(PROMIS_day_pm)=PROMIS_day_pm_gsd
58	MDS_day_mean_wpc	MDS Day score (within-person centered)	None	MDS_day_mean_wpc= MDS_day_mean-MDS_day_pm
59	MWQ_day_mean_wpc	MWQ Day score (within-person centered)	None	MWQ_day_mean_wpc= MWQ_day_mean-MWQ_day_pm
60	PROMIS_day_mean_wpc	PROMIS Day score (within-person centered)	None	PROMIS_day_mean_wpc= PROMIS_day_mean-PROMIS_day_pm
61	MDS_day_mean_wpc_z	Z MDS Day score (within-person centered)	None	MDS_day_mean_wpc_z = MDS_day_mean_wpc / MDS_day_sd
62	MWQ_day_mean_wpc_z	Z MWQ Day score (within-person centered)	None	MWQ_day_mean_wpc_z = MWQ_day_mean_wpc / MWQ_day_sd
63	PROMIS_day_mean_wpc_z	Z PROMIS Day score (within-person centered)	None	PROMIS_day_mean_wpc_z = PROMIS_day_mean_wpc / PROMIS_day_sd
64	MDS_day_pm_z	Z MDS Person Mean (grand mean centered)	None	MDS_day_pm_z = (MDS_day_pm - MDS_day_pm_gm)/MDS_day_pm_gsd
65	MWQ_day_pm_z	Z MWQ Person Mean (grand mean centered)	None	MWQ_day_pm_z = (MWQ_day_pm - MWQ_day_pm_gm)/MWQ_day_pm_gsd

66	PROMIS_day_pm_z	Z PROMIS Person Mean (grand mean centered)	None	PROMIS_day_pm_z = (PROMIS_day_pm - PROMIS_day_pm_gm)/PROMIS_day_pm_gsd
67	MDS_day_mean_lag	MDS 1-DAY LAGGED	None	
68	MWQ_day_mean_lag	MWQ 1-DAY LAGGED	None	
69	PROMIS_day_mean_lag	PROMIS 1-DAY LAGGED	None	
70	MDS_day_mean_lag_wpc	MDS 1-DAY LAGGED Within-Person Centered	None	MDS_day_mean_lag_wpc = MDS_day_mean_lag - MDS_day_pm
71	MWQ_day_mean_lag_wpc	MWQ 1-DAY LAGGED Within-Person Centered	None	MWQ_day_mean_lag_wpc = MWQ_day_mean_lag - MWQ_day_pm
72	PROMIS_day_mean_lag_wpc	PROMIS 1-DAY LAGGED Within-Person Centered	None	PROMIS_day_mean_lag_wpc = PROMIS_day_mean_lag - PROMIS_day_pm
